# *Herpesvirus pan *encodes a functional homologue of BHRF1, the Epstein-Barr virus v-Bcl-2

**DOI:** 10.1186/1471-2180-5-6

**Published:** 2005-02-03

**Authors:** Melanie Howell, Tracey Williams, Sheila A Hazlewood

**Affiliations:** 1 School of Life Sciences, Keele University, Staffordshire, UK

## Abstract

**Background:**

Epstein-Barr virus (EBV) latently infects about 90% of the human population and is associated with benign and malignant diseases of lymphoid and epithelial origin. BHRF1, an early lytic cycle antigen, is an apoptosis suppressing member of the Bcl-2 family. *In vitro *studies imply that BHRF1 is dispensable for both virus replication and transformation. However, the fact that BHRF1 is highly conserved not only in all EBV isolates studied to date but also in the analogous viruses *Herpesvirus papio *and *Herpesvirus pan *that infect baboons and chimpanzees respectively, suggests BHRF1 may play an important role *in vivo*.

**Results:**

*Herpesvirus papio *BHRF1 has been shown to function in an analogous manner to EBV BHRF1 in response to DNA damaging agents in human keratinocytes. In this study we show that the heterologous expression of the previously uncharacterised *Herpesvirus pan *BHRF1 in the human Burkitt's lymphoma cell line Ramos-BL provides similar anti-apoptotic functions to that of EBV BHRF1 in response to apoptosis triggered by serum withdrawal, etoposide treatment and ultraviolet (UV) radiation. We also map the amino acid changes onto the recently solved structure of the EBV BHRF1 and reveal that these changes are unlikely to alter the 3D structure of the protein.

**Conclusions:**

These findings show that the functional conservation of BHRF1 extends to a lymphoid background, suggesting that the primate virus proteins interact with cellular proteins that are themselves highly conserved across the higher primates. Further weight is added to this suggestion when we show that the difference in amino acid sequences map to regions on the 3D structure of EBV BHRF1 that are unlikely to change the conformation of the protein.

## Background

Apoptosis is a genetically programmed form of cell death employed to regulate cell number, spatial organisation and remove infected and damaged cell populations that may compromise the integrity of the organism [1]. Aberrant or unscheduled apoptotic responses are thought to contribute to various diseases including Alzheimer's disease, rheumatic diseases and neoplastic growth [2]. Induction of apoptosis in virally infected cells is a key weapon in the immune system's arsenal against viral attack.

Many viruses have evolved multiple strategies to counteract host-mediated apoptosis in order to facilitate infection, replication or persistence [3,4]. Epstein-Barr virus (EBV) a human γ-herpesvirus that latently infects the majority of adults, was the first virus shown to influence the survival of infected cells [5]. *In vivo*, EBV is associated with a number of benign and malignant proliferative diseases, such as infectious mononucleosis, Burkitt's lymphoma and nasopharyngeal carcinoma [6,7]. Latent infection of B cells *in vitro *leads to outgrowth of apoptosis resistant lymphoblastoid cell lines (LCLs), which phenotypically resemble activated B-cells. Although B cells can produce progeny virus, epithelial cells are also known to be permissive for viral replication. Studies have shown that EBV has at least two mechanisms of suppressing apoptosis. During latent infection latent membrane protein 1 (LMP1) is the key mediator of protection. LMP1, a CD40 mimic, up-regulates cellular Bcl-2 [8] and A20 [9] via the NFκB pathway. The virus also encodes two structural homologues of Bcl-2: BHRF1 [10,11] and the less well characterised and more controversial BALF1 [12,13] which are thought to play their major roles during lytic infection. Whilst many other viruses also have Bcl-2 homologues (v-Bcl-2s) [14], EBV and its mammalian analogues appear unique in having two v-Bcl-2s.

Despite being considered dispensable for EBV replication or cellular transformation *in vitro *[15,16], BHRF1 is extremely highly conserved in distinct geographical isolates of EBV [17], and its role *in vivo *remains unclear. BHRF1 is thought to act in lytic cycle to delay apoptosis during replication in terminally differentiating epithelium [18]. However, some EBV-positive B-cell lymphomas have been found to be positive for BHRF1 transcripts [19] and latent BHRF1 transcripts have been detected in T-cell lymphomas [20] and in EBV-transformed tightly latent B-cell lines *in vitro *[21]. Additionally, EBV BHRF1 has been shown to suppress apoptosis in lymphoid cells in response to a range of triggers including serum deprivation [11], DNA damaging agents and chemotherapeutic drugs [22] and cytokines [23]. It is possible then, that BHRF1 may play a role at some stage during latent infection, which warrants the study of this protein in B cells, the target for latent infection.

Analogues of EBV are found in the higher primate species. *Herpesvirus papio *(*H. papio*) and *Herpesvirus pan *(*H. pan*) infect baboons and chimpanzees respectively. *H. papio *encodes a BHRF1 homologue that shares 65% amino acid identity (81% similarity) with EBV BHRF1, whilst the *H. pan *BHRF1 is even more similar at 82% identity and 90% similarity [24]. *H. papio *BHRF1 has been shown to confer resistance to cisplatin induced apoptosis in human keratinocytes akin to EBV BHRF1 [25], but this study represents the first functional analysis of a primate virus BHRF1 in a lymphoid cell background. Here we show that *H. pan *BHRF1 exhibits similar anti-apoptotic activity to that of EBV BHRF1 in the human Burkitt's lymphoma cell line Ramos-BL in response to apoptosis induced by either serum withdrawal, etoposide treatment and the previously untested trigger, ultraviolet radiation. These results show that *H. pan *BHRF1 is biologically active in human B-lymphocytes *in vitro *suggesting a conserved role for BHRF1 during *in vivo *infection of B-lymphocytes.

## Results

### Construction of BHRF1 expression vectors and selection of positive clones

As the senior author has previously shown that EBV BHRF1 suppresses apoptosis in response to serum reduction in various BL cell lines [11], we initially elected to compare the abilities of *H. pan *BHRF1 and EBV BHRF1 to regulate serum reduction induced apoptosis in the EBV negative Ramos-BL cell line to ascertain whether the genes are functional, as well as structural, homologues. In order to produce expression vectors, the *H. pan *and EBV BHRF1 reading frames were amplified by PCR from template vectors pHVPanTW4 and pDH222 respectively using primers incorporating XhoI restriction sites. The PCR fragments were introduced into the pCR2.1Topo^® ^vector and the XhoI fragment was released by restriction digestion of the TopoBHRF1 constructs and cloned into the XhoI restriction site of the pcDNA4/HisMax mammalian expression vector (InVitrogen), which produces an N-terminal fusion protein with 6His and Xpress epitopes.

**Figure 1:**
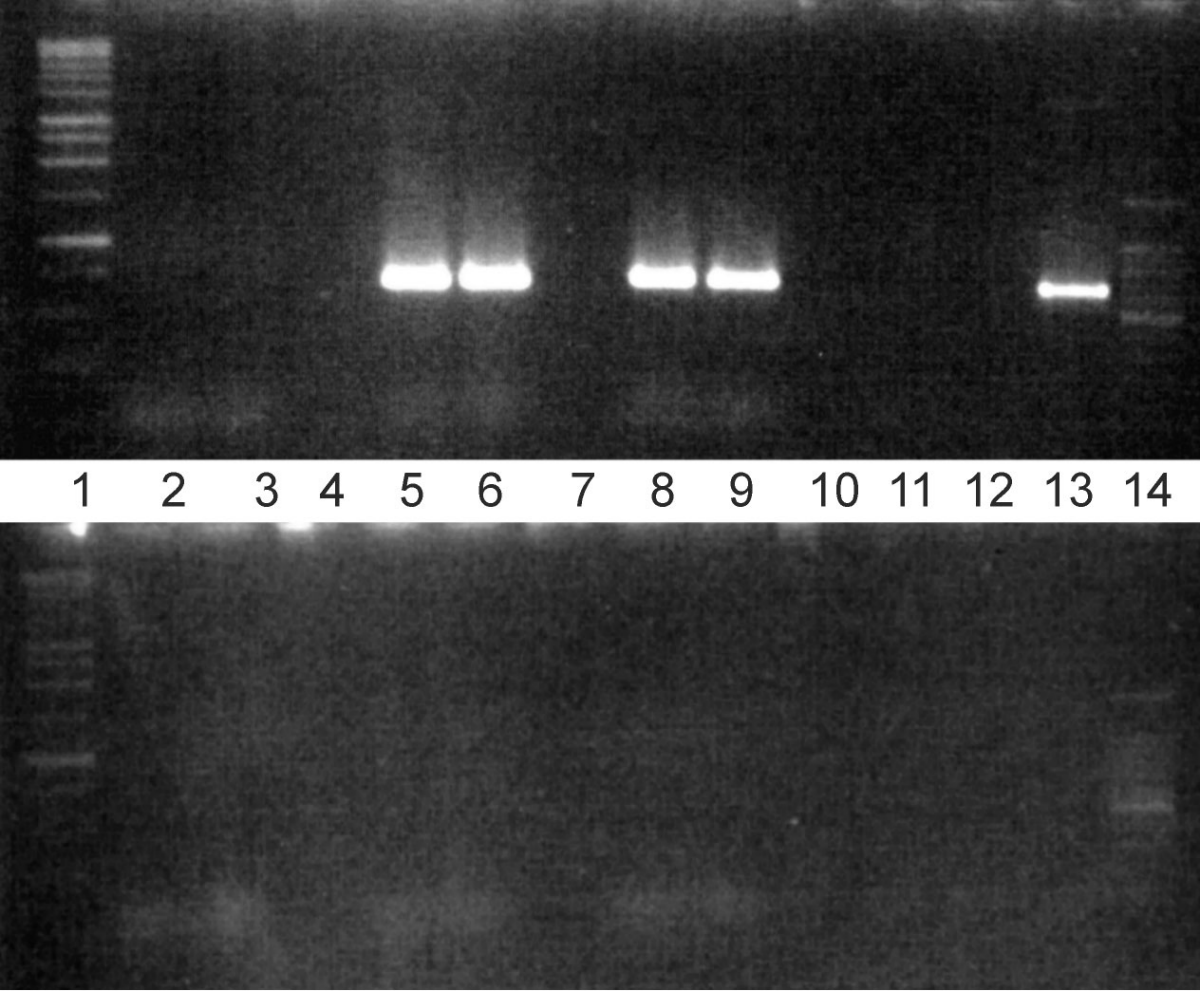
**RT-PCR analysis of transfected clones. **The upper panel shows RT-PCR analysis of transfected clones using primers designed to amplify a 655 bp fragment of the BHRF1 transcripts. From left to right samples are 1) Promega kilobase ladder, 2) vector control clone His C12, 3) vector control clone His C13, 4) Blank 5)EBV BHRF1 clone EBV 1B, 6) EBV BHRF1 clone EBV 3B, 7) Blank 8) *H. pan *BHRF1 clone Pan 4 9) *H. pan *BHRF1 clone Pan 5 10) Blank 11) Ramos-BL 12) negative RT control 13) pEBV9 14) Promega 100 bp ladder. The lower panel shows the same samples (except 13 – negative PCR control) in which the RT has been omitted in the RT step, to exclude DNA contamination in the RNA samples.

**Figure 2:**
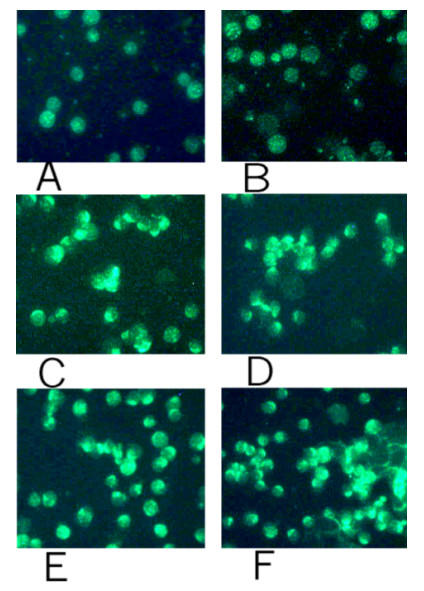
**Detection of BHRF-1 fusion protein by indirect immunofluorescence. **Cell smears were incubated with Clone His-1 monoclonal anti-polyhistidine antibody followed by FITC-conjugated goat anti-mouse antibody, viewed by fluorescence microscopy and photographed at 15× magnification (1.27 s exposure) using a digital camera. Key A – His C12; B – His C13; C – EBV 1B; D – EBV 3B; E – Pan 4 and F – Pan 5.

Ramos-BL cells were electroporated with either control vector, EBV BHRF1 or *H. pan *BHRF1 and cloned by limiting dilution in medium containing 150 μg/ml zeocin. There was no obvious difference in the numbers of drug resistant clones that were produced by transfection with the vector or either of the BHRF1 constructs. Drug resistant clones were screened by RT-PCR for BHRF1 expression and approximately 50% of the clones were shown to be expressing detectable BHRF1. The RT-PCR profiles of two clones each of the three transfected populations which were selected for further analysis, are shown in Figure 1. RT negative samples were included (lower panel) to rule out DNA contamination of RNA samples. Expression of the fusion proteins was confirmed by Western blot analysis (not shown) and by indirect immunofluorescence using an antibody directed against the 6His epitope (Figure 2). Some diffuse fluorescence is seen in the His C controls (A and B), but this is to be expected as the fusion part of the protein is at the amino terminus and the vector therefore can produce a small protein containing the 6His epitope. There is a distinctive crescent-like pattern of fluorescence in clones transfected with the EBV BHRF1 (C and D) and *H. pan *BHRF1 (E and F), indicative of BHRF1 expression.

### Determination of sensitivity to serum withdrawal induced apoptosis

**Figure 3:**
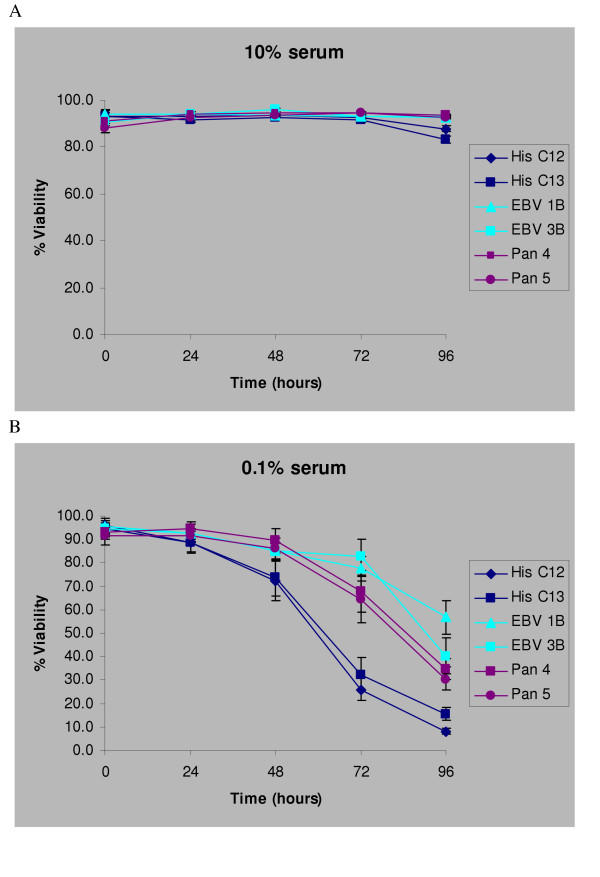
**Determination of apoptosis levels in reduced serum conditions. **The percentage viability of EBV and *H. pan *BHRF1 Ramos-BL transfectants compared to vector controls in medium containing A) 10% serum or B) 0.1% serum over a 4 day period. The % viability was determined daily by acridine orange fluorescence. The data plotted are the means three experiments carried out in triplicate ± standard error. Statistical analysis was performed using ANOVA and Tukey's pairwise comparison on data normalised by arcsine transformation.

To determine the sensitivity of the transfected clones to the induction of apoptosis by serum reduction, EBV clones EBV 1B and EBV 3B, *H. pan *clones Pan 4 and Pan 5 and BHRF1 null vector control clones His C12 and His C13 were seeded in medium containing either 10% or 0.1% serum and the percentage of apoptotic cells was determined at 24-hourly intervals, using acridine orange to distinguish between apoptotic and viable cell nuclei. Necrotic nuclei that appeared after several days due to secondary necrosis of cells already lost by apoptosis were excluded from the counts. Apoptotic death was also confirmed by DNA laddering (not shown). We found that when maintained in medium containing 10% serum (Figure 3A), both the EBV BHRF1 clones exhibited a slight drop in viability after 48 hours and thereafter viabilities increased to above 90%. A similar drop in viability was seen in the two vector clones, but after an increase at 72 hours, both clones began to show a decline in viability by 96 hours. Both the *H. pan *BHRF1 expressing clones, maintained viability above 90% throughout the 96 hours period. In contrast, the BHRF1 null vector controls showed higher rates of cell death compared to either the EBV or *H. pan *BHRF1 expressing clones from 24 hours onwards in medium containing 0.1 % serum (Figure 3B). After 96 hours in culture, approximately 15% viability was observable in the BHRF1 null vector controls whilst the viability of the BHRF1 clones ranges from 35 to 49%. Clone EBV IB was the most resistant to apoptosis induced by serum deprivation with 49% viability after 96 hours. Two-way ANOVA and Tukey's pairwise analysis of arcsine transformed data showed that at all time points from 48 hours onwards there were significant differences (p < 0.001) between the BHRF1 expressing clones and the vector control clones, but not between the Pan and EBV BHRF1 clones, except at 96 hours when clone EBV 1B showed significantly higher viability than all other clones (eg p = 0.0001 when compared to EBV 3B). Therefore our data indicates that 0.1% serum is sub-optimal for survival of Ramos-BL since a high degree of apoptotic cells are observable at this concentration. Clearly however, both EBV and *H. pan *BHRF1 expression were able to delay the apoptotic response, and thus promote the survival of these cells which would otherwise undergo apoptosis.

### Determination of sensitivity to etoposide induced apoptosis

**Figure 4:**
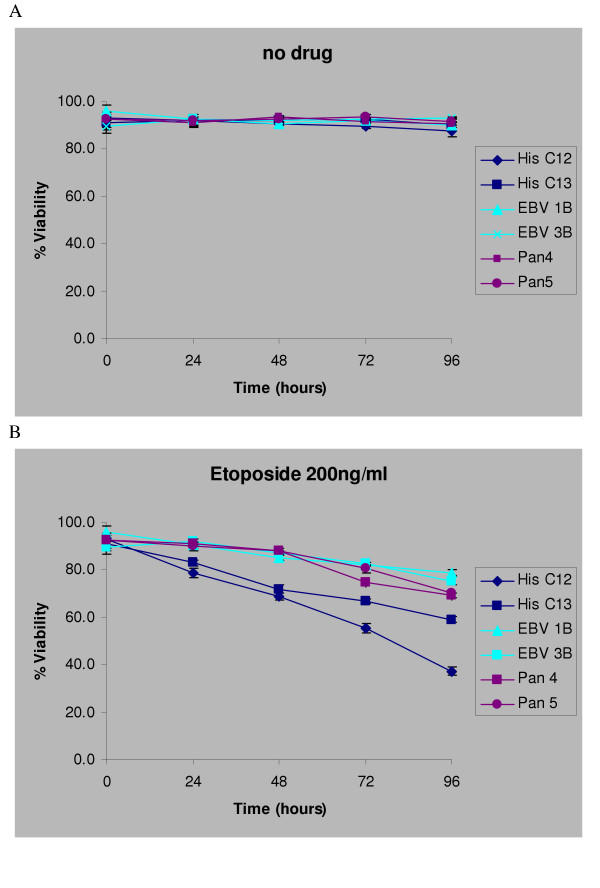
**Determination of apoptosis levels in etoposide treated cells. **The percentage viability of EBV and *H. pan *BHRF1 Ramos-BL transfectants compared to vector controls in medium containing A) DMSO control or B) 200 ng/ml etoposide over a 4 day period. The % viability was determined daily by acridine orange fluorescence. The data plotted are the means three experiments carried out in triplicate ± standard error. Statistical analysis was performed using ANOVA and Tukey's pairwise comparison on data normalised by arcsine transformation

We then investigated whether or not the proteins could regulate apoptosis induced by the DNA damaging agent etoposide. The transfected clones were seeded in medium containing either no etoposide or 200 ng/ml etoposide. The percentage of viable versus apoptotic cells was determined every 24 hours by acridine orange fluorescence. We found that, in the absence of etoposide, viability was maintained at above 90% in all clones (Figure 4A). In the presence of etoposide (Figure 4B), both the control clones showed a steady decline in viability from 24 hours onwards, with clone His C13 maintaining higher viability than His C12, whilst the BHRF1 clones remained viable for 48 hours before starting to show a decline in viability. Two way ANOVA and Tukey's pairwise comparisons of arcsine transformed data showed that the differences between the control and BHRF1 clones were significantly different from 24 hours onwards. Whilst clone His C13 was significantly different to the BHRF1 clones at all time points, it was also significantly different to His C12 at the 72 (p = 0.359) and 96 (p = 0.002) hour time points. There were no significant differences between the EBV and Pan BHRF1 clones.

### Determination of sensitivity to ultraviolet induced apoptosis

**Figure 5:**
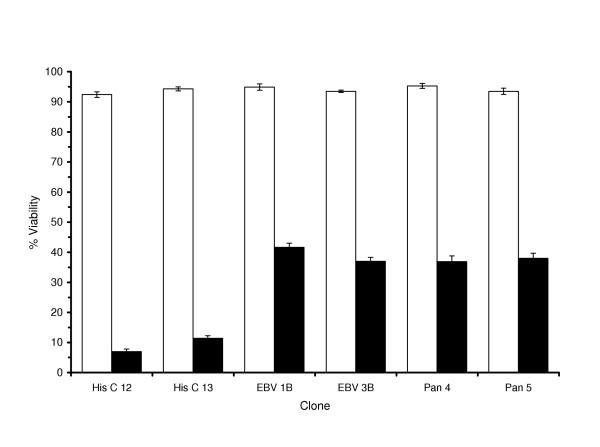
**Determination of apoptosis levels in UV irradiated cells. **The effect of 30 Jm^-2 ^UV irradiation (solid bars) on EBV BHRF1 and *H. pan *BHRF1 transfected Ramos-BL clones compared to vector controls. Cell viability was assayed after 24 hours by acridine orange fluorescence. The data plotted are the means three experiments carried out in triplicate ± standard error. Statistical analysis was performed using ANOVA and Tukey's pairwise comparison on data normalised by arcsine transformation

Ultraviolet radiation has been shown to induce apoptosis in other cell lines within a 24 hour period. Having first determined the UV conditions necessary to induced apoptosis in the parent cell line, we then exposed the transfected clones to UV radiation at 30 Jm^-2^. Acridine orange counts were taken after 24 hours and the results are shown in figure 5. Statistical analysis was performed on both the transformed and untransformed data. The untransformed data is shown in Figure 4, as the error bars were so small in the transformed data that they were not visible. The results are highly significant with p < 0.000001.

### Mapping the amino acid changes on to the 3D structure

**Figure 6:**
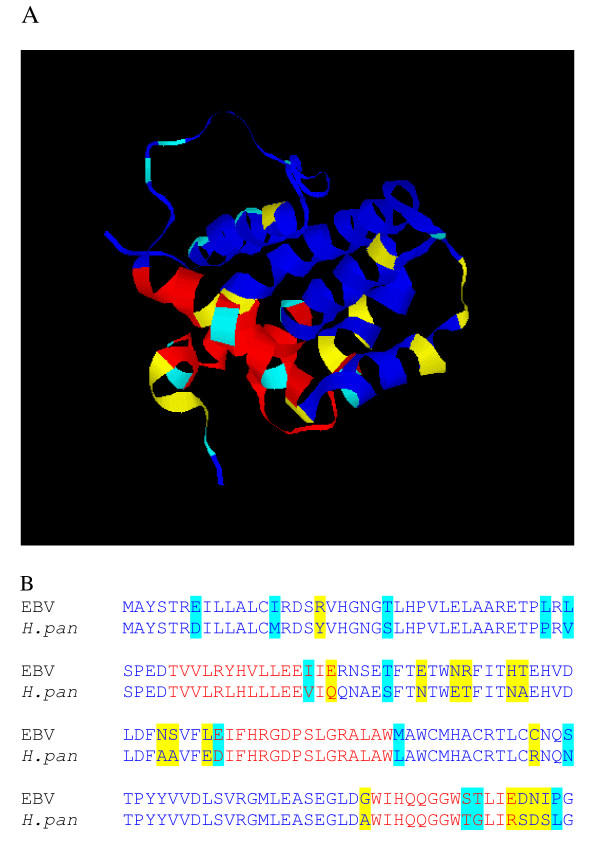
**Mapping of amino acid changes in *H. pan *BHRF1 on the EBV BHRF1 structure. **The structure of the first 160 amino acids of EBV BHRF1 was visualised by importing the structural data file (1q59.pdb) into RasMol v2.6, which converts the structural data into a 3 D image (Figure 6A) Using the RasMol edit commands we have highlighted the BH domains in red, whilst the remainder of the protein is shown in dark blue. Conservative replacements in the *H. pan *protein are highlighted in light blue and non-conservative replacements are shown in yellow. For comparison the aligned amino acid sequenced are shown in Figure 6B, using the same colour coding as in Figure 6A.

During the course of this study, the structure of BHRF1 was solved [26]. Using RasMol, v2.6, the freely available molecular visualisation software created by Roger Sayle, we have produced a 3D image of BHRF1, highlighting the regions where the EBV and *H. pan *proteins differ, as shown in Figure 6A. Non-conservative changes, shown in yellow, tend to be found at the end of helices, whereas conservative replacements tend to be within the helices. There are changes within the known functional BH domains (shown in red). The aligned primary amino acid sequences, with the BH domains and changes highlighted using the same colour scheme are shown in Figure 6B.

## Discussion

In this study we present evidence that the BHRF1 protein encoded by *H. pan *is functionally homologous to the analogous protein in the EBV in human B cells. BHRF1 is one of two Bcl-2 homologues encoded by EBV. Previously the EBV BHRF1 protein has been shown to protect transfected B-cells from apoptosis [11] and delay the differentiation of epithelial cells *in vitro *[18]. The primate virus analogues of BHRF1 have now been cloned by us and others [24,25] and have been shown to be highly homologous in their primary structure both at the DNA and protein level. BHRF1 is normally expressed at high levels in during lytic replication in epithelial cells and the first functional studies of a primate BHRF1, namely the baboon virus BHRF1, were carried out in a keratinocyte system. Whilst a role for BHRF1 during latent infection has not been proven, circumstantial evidence that BHRF1 transcripts are found in certain lymphoid malignancies and that BHRF1 can suppress apoptosis in lymphoid cells suggests that BHRF1 has the potential to contribute to malignant transformation. Whilst infection of B cells is largely latent, lytic replication in B cells also plays an important role in EBV's lifecycle by providing virus to re-infect epithelial cells, ensuring the continued shedding of virus in the oropharynx. We therefore decided to investigate whether the functional homology extended to a lymphoid cell background by comparing EBV BHRF1 with the previously uncharacterised BHRF1 from *H. pan*. We used 3 triggers known to induce apoptosis at differing rates. Serum withdrawal is a slow trigger, with low levels of apoptosis for 48 hours followed by a precipitous drop in viability. The kinetics of etoposide activity are very different with a steady decline in viabilty over the 4 day period, whilst UV irradiation (never previously reported for BHRF1) induces apoptosis within hours. The EBV and *H. pan *BHRF1s afforded significant protection against the induction of apoptosis by all three triggers, compared to the vector controls. There was no statistical difference between the EBV and *H. pan *BHRF1s, (except for clone EBV1B at 96 hours in 0.1% serum), suggesting that *H. pan *BHRF1 is indeed a true functional homologue of EBV BHRF1. The difference between clone EBV1B and the other BHRF1 clones at 96 hours in low serum could possibly be attributed to slightly higher levels of BHRF-1 expression in this clone, although it is difficult to quantify absolute expression levels from the fluorescence images shown in Figure 2. Clone His C13 maintained higher viability than clone His C12 against all three triggers and when exposed to etoposide this is statistically significant from 72 hours onwards. This possibly reflects genetic changes accumulated by the clones during selection and passage and highlights the importance of including more than one clone in assays such as these.

When one looks more closely at the regions of the protein that differ between the human and chimpanzee viruses (Figure 6), it is perhaps not surprising that the two proteins are functional homologues, despite the changes in the amino acid sequence. The non-conservative changes are almost always at the end of a helix where they are least likely to disrupt the overall structure of the protein, whilst the conservative changes are mainly within the helices or loops. Notably there are some changes within the BH domains, known to be critical for the function of Bcl-2 family proteins, which are clearly tolerated in terms of conservation of function. None of the observed changes are likely to significantly alter the conformation of the protein, highlighting the fact that functional conservation of BHRF1 is subject to 3D structural constraints. These observations emphasise the importance of combining structural information with homology studies such as this one to identify regions of the protein that need to be conserved to retain structural integrity. That the two proteins appear to be so similar at the 3D level implies they probably interact with cellular proteins that are themselves highly conserved in the two species. There are only a few reports of proteins known to interact with BHRF1, including PRA1 [27] and although some studies have shown interactions with several Bcl-2 family proteins [28,29], the reported differences in the 3D structures of BHRF1 and Bcl-2 (namely the lack of a binding groove for pro-apoptotic Bcl-2 family members) [26] suggests that the anti-apoptotic activity of BHRF1 does not exactly parallel that of Bcl-2. This warrants further study into the viral and cellular proteins which interact with BHRF1, not only to yield more insight into the interplay between virus and host, but also to further our knowledge of the fundamental mechanisms of apoptosis.

## Conclusions

Our results are the first demonstration of functional homology between a primate BHRF1 (*H. pan*) and EBV BHRF1 in a human lymphoid cell background. Both proteins protect against apoptosis induced by two previously described triggers and also a new trigger, UV radiation, against which BHRF1 has never been reported to provide protection. Comparison of the EBV and *H. pan *proteins at the 3D structural level reveals that none of the changes is likely to significantly alter the structure of the protein.

## Methods

### Construction of expression vectors

The 573 bp coding sequences of EBV BHRF1 and *H. pan*BHRF1 were amplified by PCR from pDH222 [11] and pHVPanTW4 [24] respectively using primers which incorporated *Xho*I sites flanking the translational start and stop codons. We used Promega's PCR mastermix with 50 pmol each primer. The sequence of the forward primer was TTGCAGCTCGAGATGGCCTATTC and the reverse primer sequence was GAAAATCTCGAGATTAGTGTCTTCC. The PCR parameters were 35 cycles of 95°C (30 sec), 55°C (30 sec) and 72°C (60 sec). The PCR products were introduced by Topo TA cloning into pCR2.1-Topo^® ^(Invitrogen) and the *XhoI *fragments were released by digestion with *XhoI *and cloned into the *Xho*1 site of pcDNA4/HisMaxC (Invitrogen). The plasmids were sequenced to verify that the sequences had joined in frame and contained no PCR induced errors.

### Transfection of Ramos-BL cells

6 × 10^6 ^Ramos-BL cells were independently transfected with 20 μg endotoxin-free EBV BHRF1, *H. pan*BHRF-1 or vector control constructs (pEBV9, pPan4 and pcDNA4/HisMax respectively) by electroporation in a BioRaD Gene Pulser II at 0.45 kV, 125 μF. The cells were seeded in 96-well plates at a density of 5 × 10^4 ^cells/ well in RPMI 1640 supplemented with 10% FetalClone III calf serum (HyClone) 2 mM L-glutamine (Sigma) 0.4% gentamycin (Sigma) and 200 μg/ml Zeocin (Invitrogen). Wells showing outgrowth of drug resistant cells were cloned by limiting dilution and clones were expanded into 2 ml cultures and maintained in medium containing 150 μg/ml Zeocin. All cultures were fed twice weekly by replacing 90% of the culture with fresh medium.

### RT-PCR

Total RNA was purified from 5 × 10^6 ^cells using the RNA Safekit (Q Biogene) according to manufacturers instructions. Reverse transcription of 100 ng RNA with oligo dT primer using the Improm II kit (Promega) in a total volume of 20 μl according to manufacturer's instructions was followed by PCR (parameters as above) using 5 μl of the template cDNA and 50 pmols each of the forward and reverse primers CTGTACGACGATGACGATAAG and GTGTCTTCCTCTGGAGATA respectively, to amplify a 655 bp product.

### Indirect immunofluorescence

Cells were suspended in PBS and spotted onto multiwell glass slides and air dried for 60 minutes. Slides were fixed for 20 minutes in methanol at -20°C followed by 5 minutes in acetone at -20°C. The cell spots were rehydrated in 20% normal rabbit serum (in PBS) for 20 minutes then incubated with a 1 in 500 dilution of the monoclonal anti-poly histidine antibody (Clone HIS-1, Sigma-Aldrich) for 1.5 hours at room temp. After three 5 minute washes with PBS, the cells were incubated with a 1 in 50 dilution of FITC goat anti-mouse IgG (Biomeda) for 1 hour at room temp. Thereafter the slides were washed thrice in PBS, a drop of DABCO was added and coverslips were placed over the cell spots. The cells were viewed with by fluorescence microscopy and photographed with a Leica digital camera using a 1.27 sec exposure time and 15× magnification.

### Apoptosis assays

For the serum reduction assay cells were washed 3 times in PBS and then 0.5 × 10^6 ^cells (BHRF1 or vector clones) were seeded in either medium containing 10% serum or 0.1% serum and the percentage of viable versus apoptotic cells was determined by acridine orange flourescence at 24 hour intervals. For the etoposide assays, 0.5 × 10^6 ^cells (washed 3 times) were seeded in either routine medium containing DMSO (equivalent to the volume added to the etoposide samples) or medium containing 200 ng/ml etoposide (diluted from a 1 mg/ml solution in DMSO) and the % of apoptotic cells versus viable cells was determined every 24 hours by acridine orange fluorescence. For the UV induction assays, 1 × 10^6 ^cells in 10 ml of medium were placed in a Petri dish and exposed to 30 Jm^-2 ^UV. The cells were then pelleted by centrifugation and resuspended at 2 × 10^5^cells/ml in medium and returned to culture flasks.

Each clone was tested in triplicate and each experiment was repeated 3 times.

### Acridine orange fluorescence

10 μl acridine orange (100 μg/ml) was added to 100 μl of cell culture containing the individual clones. At least 200 cells were counted using a haemocytometer at 15 × magnification and the number of viable versus apoptotic cells in the population noted.

## List of abbreviations

EBV – Epstein Barr virus, *H. pan *– *Herpesvirus pan*, PBS phosphate buffered saline, PCR – polymerase chain reaction, UV – ultraviolet, v-Bcl-2 – viral Bcl-2 homologue

## Authors' contributions

MH did the RT-PCR and UV assay, TW constructed the expression vectors and carried out some of the statistical analyses and SAH produced the transfected clones, carried out the serum withdrawal and etoposide assays, some of the statistical analyses and did the structural comparisons.
